# Supplementary material for: Fast and reversible neural inactivation in macaque cortex by optogenetic stimulation of GABAergic neurons
Source: eLife. 2020 May 26;9:e52658. doi: 10.7554/eLife.52658 (PMC7329331; doi:10.7554/eLife.52658)
Supplement: Transparent reporting form [file elife-52658-transrepform.docx]

***eLife’s* transparent reporting form**

We encourage authors to provide detailed information *within their submission* to facilitate the interpretation and replication of experiments. Authors can upload supporting documentation to indicate the use of appropriate reporting guidelines for health-related research (see [EQUATOR Network](http://www.equator-network.org/%20)), life science research (see the [BioSharing Information Resource](https://biosharing.org/)), or the [ARRIVE guidelines](http://www.plosbiology.org/article/info:doi/10.1371/journal.pbio.1000412) for reporting work involving animal research. Where applicable, authors should refer to any relevant reporting standards documents in this form.

If you have any questions, please consult our Journal Policies and/or contact us: [editorial@elifesciences.org](mailto:editorial@elifesciences.org).

**Sample-size estimation**

- You should state whether an appropriate sample size was computed when the study was being designed
- You should state the statistical method of sample size computation and any required assumptions
- If no explicit power analysis was used, you should describe how you decided what sample (replicate) size (number) to use

Please outline where this information can be found within the submission (e.g., sections or figure legends), or explain why this information doesn’t apply to your submission:

The sample size was 3 rhesus macaque monkeys. Of those, one was exclusively used for obtaining histological data and the other two were used for obtaining neurophysiological and behavioral data. It is customary in primate neurophysiology and behavior studies to confirm that results are similar in two animals. All animal procedures conformed to NIH guidelines and were approved by the Institutional Animal Care and Use Committee at the University of Washington.

**For more details please see MATERIALS & METHODS (Page 23)**

**Replicates**

- You should report how often each experiment was performed
- You should include a definition of biological versus technical replication
- The data obtained should be provided and sufficient information should be provided to indicate the number of independent biological and/or technical replicates
- If you encountered any outliers, you should describe how these were handled
- Criteria for exclusion/inclusion of data should be clearly stated
- High-throughput sequence data should be uploaded before submission, with a private link for reviewers provided (these are available from both GEO and ArrayExpress)

Please outline where this information can be found within the submission (e.g., sections or figure legends), or explain why this information doesn’t apply to your submission:

**Page 5:** The **histological** results reported in this study (**Figure 1, Figure 1-Figure Supplement 1**) are based on the analyses of two cortical sections from Monkey 1. The number of cells labeled via different antibodies are mentioned in the figure legends.

**Page 5-6:** The **neurophysiological** results reported in this study (**Figure 2, Figure 3, Figure 2-Figure Supplement 1**) were collected from Monkey 2 and Monkey 3 while they performed a visually engaging behavioral task (Gabor contrast detection task). A total of 56 units (25 from Monkey 2 and 31 from Monkey 3, respectively) contributed to this study. The units were selected because they were modulated by optical stimulation as assessed with audio monitoring online. For each of these units, we report the average firing rate over 30 repeats of optical stimulation.

**Page 15:** We report data from 1 single-unit from Monkey 2 during a passive fixation task (**Figure 9**). We collected the neuronal spikes in response to sinusoidal optical stimulation to different frequencies (2-254 Hz). We report the average responses over 2-4 repeats of optical stimulation.

**Page 7:** We report **behavioral data** from a **visually guided saccade task** from Monkey 2 and Monkey 3 **(Figure 4, Figure 4-Figure Supplement 1**). Data from 17 sessions (10 from Monkey 2 and 7 from Monkey 3, respectively) contributed to this study. During these sessions, the neural activity was modulated by optical stimulation. Behavior was impacted during optical stimulation for most sessions (10/10 for Monkey 2 and 5/7 from Monkey 3).

**Page 8-10:** We report **behavioral data** from a **Gabor contrast detection** **task** from Monkey 2 and Monkey 3 **(Figure 5, Figure 6, Figure 6-Figure Supplement 1, Figure 7, Figure 8**). Data from 23 contributed to this study sessions (11 from Monkey 2 and 12 from Monkey 3, respectively). During these sessions, neural activity was modulated by optical stimulation. Most sessions showed significant change in behavior with optical stimulation (10/11 for Monkey 2 and 11/12 for Monkey 3, respectively).

**Page 9:** We analyzed the **link between neural activity and behavior** while the monkeys performed the Gabor contrast detection task. We report data from these session in **Figure 8-Figure Supplement 1**. We plot two subfigures 1) including all the 56 sites 2) data from one single session to study the impact of laser power keeping other covariates constant. Please see Page 8 for more details.

**Statistical reporting**

- Statistical analysis methods should be described and justified
- Raw data should be presented in figures whenever informative to do so (typically when N per group is less than 10)
- For each experiment, you should identify the statistical tests used, exact values of N, definitions of center, methods of multiple test correction, and dispersion and precision measures (e.g., mean, median, SD, SEM, confidence intervals; and, for the major substantive results, a measure of effect size (e.g., Pearson's r, Cohen's d)
- Report exact p-values wherever possible alongside the summary statistics and 95% confidence intervals. These should be reported for all key questions and not only when the p-value is less than 0.05.

Please outline where this information can be found within the submission (e.g., sections or figure legends), or explain why this information doesn’t apply to your submission:

(For large datasets, or papers with a very large number of statistical tests, you may upload a single table file with tests, Ns, etc., with reference to sections in the manuscript.)

**Page 5:** Most mCherry-positive neurons co-expressed parvalbumin (**37/41 in Figure 1 and 468/543 in Figure 1-Figure Supplement 1**), a marker for 75% of GABAergic neurons in macaque V1. This high co-expression suggests a significant bias towards parvalbumin neurons (p<0.005, binomial test).

**Page 5:** 56 units were modulated by optical stimulation (p<0.06, Mann Whitney U test; **Figure 2C**). Of these, 36 were excited and 18 were suppressed by optical stimulation.

**Page 5:** Most units were visually driven (46/56). Of these, 19 attained statistical significance (p<0.05, Mann Whitney U test).

**Page 6:** Post-laser firing rates were lower than pre-laser firing rates at most sites (16/18, p<0.05, Wilcoxon signed rank test)

**Page 7**: In the **visually guided saccade task**, the saccade end points tended to be closer to the target on control trials than on laser trials when the target appeared inside the receptive fields of illuminated neurons (p<0.002 for Monkey 2, p<0.03 for Monkey 3, Wilcoxon signed rank test). When target appeared outside, no difference in behavior was observed between control and laser trials (p=0.92 for Monkey 2, p=0.38 for Monkey 3, Wilcoxon signed rank test).

**Page 7:** Analysis of saccade latencies was performed for the visually guided saccade task. Saccade latencies were greater on laser trials than on control trials when targets were inside (p<0.0001 for Monkeys 2 & 3, Mann Whitney U test) but not when targets were outside the receptive fields of the illuminated neurons (p>0.4 for Monkeys 2 & 3, Mann Whitney U test). The population results from this task is reported in **Figure 4-Figure Supplement 1**.

**Page 7-9:** For the Gabor contrast detection task, behavior was quantified using 4 variables: hits (H), misses (M), false alarms (FA) and correct rejects (CR). In almost every session (10/11 in Monkey 2 and 11/12 in Monkey 3), the proportion of hits on control trials was significantly greater than on laser trials (p<0.05; binomial test for equality of proportions; **Figure 7A-B**).

**Page 7-9**: The four variables (H,M,CR,FA) were condensed into a single scalar variable (*d’*) that was operationally defined as the sensitivity index. We confirmed that *d’* on control trials was not affected by the changes in laser power (Pearson’s r=-0.37, p=0.41) and the performance on controls trials was unaffected by the interleaved laser trials (p=0.11, likelihood ratio tests on the joint and individual Weibull fits). *d’* on laser trials was lower than *d’* on control trials in most sessions (10/11 in Monkey 2 and 12/12 in Monkey 3; **Figure 7C-D**). Difference in *d’* between laser and control trials varied little as function of block number within a session (Pearson’s r=0.25, p=0.45; **Figure 8C**). Analysis of individual sessions yielded the same result (p=0.57, t test on the regression slopes). The behavioral effect did not differ between the early and late trials (p=0.79, t test; **Figure 8D**).

**Page 9:** Neural activity was tightly coupled with behavior in one experimental session (Spearman’s r=0.61, p=0.17). Pooling the data across all blocks of trials reduced this correlation (Spearman’s r=0.16, p=0.23). This result is reported in **Figure 8-Figure Supplement 1**.

**Group allocation**

- Indicate how samples were allocated into experimental groups (in the case of clinical studies, please specify allocation to treatment method); if randomization was used, please also state if restricted randomization was applied
- Indicate if masking was used during group allocation, data collection and/or data analysis

Please outline where this information can be found within the submission (e.g., sections or figure legends), or explain why this information doesn’t apply to your submission:

Samples were not groups in this study. The three monkeys used in this study contributed different types of data:

1)	Histology: Monkey 1

2)	Neurophysiology: Monkey 2 & 3

3)	Behavior: Monkey 2 & 3

**For more details please see MATERIALS & METHODS (Page 23) and RESULTS (Page 4)**

**Additional data files (“source data”)**

- We encourage you to upload relevant additional data files, such as numerical data that are represented as a graph in a figure, or as a summary table
- Where provided, these should be in the most useful format, and they can be uploaded as “Source data” files linked to a main figure or table
- Include model definition files including the full list of parameters used
- Include code used for data analysis (e.g., R, MatLab)
- Avoid stating that data files are “available upon request”

Please indicate the figures or tables for which source data files have been provided:

Data are available at <https://github.com/horwitzlab>.
